# A Robust Multivariate Thresholding Function for Sparse and Biomedical Signal Reconstruction

**DOI:** 10.3390/s26113595

**Published:** 2026-06-05

**Authors:** Hayat Ullah, Sunil Gaire, Corey A. Graves

**Affiliations:** Department of Electrical and Computer Engineering, North Carolina Agriculture and Technical State University, Greensboro, NC 27411, USA; skgaire@ncat.edu

**Keywords:** ECG sensors, signal denoising, sparse signal processing, Gaussian mixture model, thresholding function, biomedical sensing

## Abstract

This paper presents a computationally efficient Multivariate Mixture Model Thresholding (MMMT) technique for sparse signal denoising and recovery, with the goal of improving data quality in modern sensing and biomedical systems. The proposed method extends classical thresholding approaches by modeling nonzero signal coefficients using a multivariate Gaussian mixture prior, thereby capturing cross-channel and intercomponent dependencies commonly observed in multi-sensor and physiological signals. The thresholding rule is analytically derived through maximum a posteriori (MAP) estimation within a majorization–minimization (MM) optimization framework, while the associated model parameters are adaptively estimated using an expectation–maximization (EM) algorithm. Experimental results on noisy sinusoidal signals and synthetic ECG data demonstrate that MMMT consistently achieves higher correlation with ground-truth signals and improved preservation of pulse amplitude and morphological characteristics compared with benchmark methods, including the $l_{1}$-fused lasso and convex–non-convex (CNC) fused lasso. Quantitative evaluations based on correlation metrics, signal-to-noise ratio (SNR), and peak signal-to-noise ratio (PSNR) further confirm the effectiveness of the proposed approach. Owing to its scalability, robustness, and strong statistical interpretability, MMMT provides a promising framework for real-time ECG signal enhancement. Although the proposed framework is general and can be adapted to other biomedical modalities such as EEG, CT, and MRI, experimental validation in this study is limited to ECG signals.

## 1. Introduction

Signal denoising and recovery remain fundamental challenges in signal processing, particularly when reconstructing signals from noisy, limited, or undersampled measurements. These challenges are especially critical in biomedical signal processing, where the preservation of clinically relevant waveform morphology is essential. The amplitude and shape of electrocardiogram (ECG) pulses are as important as the suppression of noise [1,2,3]. In practical application settings, including wearable health monitoring, ambulatory ECG acquisition, and real-time clinical decision support systems, denoising algorithms must operate reliably under severe noise, motion artifacts, and limited sampling conditions. Classical reconstruction techniques based on least-squares minimization and the $l_{2}$-norm are computationally efficient; however, they typically perform poorly under undersampling conditions and fail to promote sparsity, resulting in suboptimal recovery and increased computational burden in these application-driven scenarios.

Although the $l_{0}$-norm directly promotes sparsity, the associated optimization problem is NP-hard and computationally intractable in most practical scenarios. As a result, the $l_{1}$-norm has become a widely adopted convex surrogate due to its ability to shrink small coefficients toward zero and encourage sparse representations. More generally, $l_{p}$-norms with 0<p<1 have been explored to enhance sparsity promotion by imposing stronger penalties on nonzero coefficients at the cost of non-convexity and increased algorithmic complexity. In application-oriented biomedical systems, this trade-off between reconstruction accuracy, computational efficiency, and robustness remains an open challenge, particularly for real-time or resource-constrained deployments.

More recently, significant attention has been paid to denoising biomedical signals and images, including ECG, EEG, and MRI. Adaptive and nonconvex thresholding strategies have been shown to improve noise suppression while partially alleviating amplitude shrinkage [4,5,6]. Recent studies have also explored adaptive wavelet and transform-domain denoising strategies tailored to biomedical sensing applications [7,8,9]. In particular, recent work has proposed layer-dependent and data-adaptive thresholding functions for ECG and physiological signal denoising, demonstrating improved robustness under high-noise conditions and multimodal sensing environments [10,11,12]. Despite these advances, designing denoising frameworks that simultaneously ensure morphological fidelity, interpretability, and scalability across diverse types of biomedical signals remains a key open problem.

Beyond wavelet-based methods, alternative signal decomposition approaches, such as empirical mode decomposition (EMD), variational mode decomposition (VMD), and their hybrids have been investigated for the denoising of biomedical signals, particularly for ECG and EEG signals [13,14,15]. These methods are attractive for nonstationary signals commonly encountered in practical biomedical settings, including long-term EEG monitoring and ambulatory ECG acquisition. However, their performance is often sensitive to mode mixing, parameter selection, and signal-dependent decomposition behavior, which can limit robustness and reproducibility in real-world clinical applications. In parallel, low-rank and sparse representation frameworks have been employed to exploit global signal structure for biomedical image and signal denoising [16,17], while effective in controlled settings, such approaches may suffer from high computational complexity and reduced adaptability when deployed in real-time or resource-constrained biomedical systems.

In addition, data-driven approaches such as independent component analysis (ICA) [18], ensemble neural networks [19], and deep learning–based models [20,21] have demonstrated promising denoising performance for ECG signals. These techniques are particularly attractive for large-scale data analysis and automated diagnosis pipelines. However, learning-based methods often require large labeled datasets, incur high computational cost, and may distort clinically important waveform features, thereby limiting their interpretability, generalizability, and robustness in real-world biomedical sensing applications [22]. Recent surveys and comparative studies further highlight the trade-off between denoising accuracy and morphological fidelity in deep and hybrid models, motivating the need for interpretable, model-based alternatives that can operate reliably under limited data and strict clinical constraints [23].

Sparse recovery methods based on the $l_{1}$-norm remain widely used due to their convexity and computational efficiency; however, they are known to systematically underestimate large-amplitude signal components [24]. Moreover, the $l_{1}$-norm does not form a tight convex envelope of the $l_{0}$-norm [25], leading to inherent trade-offs between sparsity enforcement and reconstruction fidelity. To address these limitations, convex–nonconvex (CNC) fused lasso formulations have been proposed [26], achieving improved performance in piecewise-constant signals and ECG denoising. Nevertheless, CNC-based methods remain highly sensitive to regularization parameter selection and may degenerate to soft thresholding under certain conditions, thereby reintroducing amplitude suppression. These limitations highlight the need for denoising frameworks that balance robustness, interpretability, and amplitude preservation across diverse biomedical signal types.

Furthermore, in high-noise environments, conventional digital filtering and QRS detection algorithms [27] often suffer from elevated false-positive rates, particularly in wearable, ambulatory, and long-term ECG monitoring scenarios [3]. In such application settings, signals are frequently corrupted by motion artifacts, baseline wander, and nonstationary noise, while computational and energy constraints limit the use of complex processing pipelines. These challenges motivate the development of robust, sparsity-aware denoising frameworks that can reliably preserve clinically meaningful waveform morphology while effectively suppressing noise across diverse acquisition conditions.

**Figure 1:**
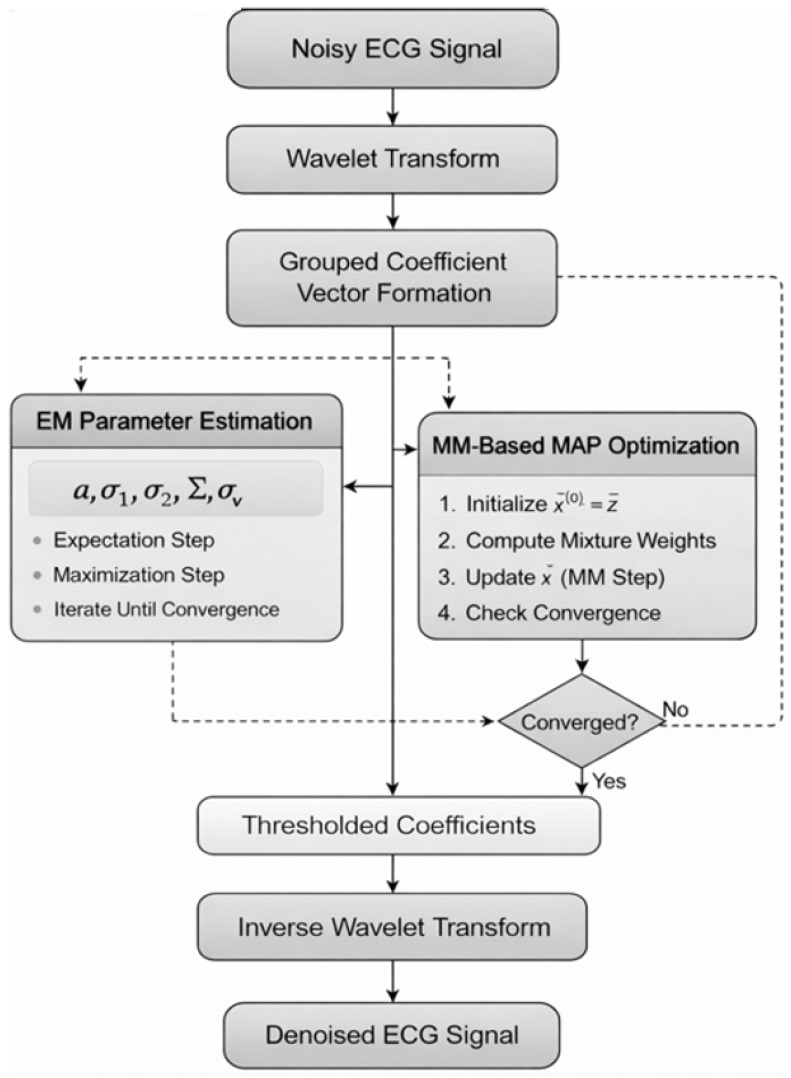
Workflow diagram of the proposed MMMT framework. EM is used to estimate mixture parameters, and the MAP estimate is computed via an MM-based iterative update in the transform domain.

Figure 1 summarizes the main steps of the proposed MMMT pipeline. After a transform-domain representation is obtained, neighboring coefficients are grouped to enable multivariate modeling. The model parameters are estimated using EM and the MAP estimate is computed using an iterative update based on MM until convergence.

In this work, we consider the problem of estimating an underlying sparse signal in a transformed domain commonly used in biomedical signal processing, such as wavelet or time–frequency representations [7,9]. Such representations are widely adopted in practical systems due to their ability to compactly capture transient structures, including ECG QRS complexes and EEG spikes. The noisy observation model is given by

(1) $$\begin{array}{c} z=Ax+\nu , \end{array}$$

where *z* denotes the observed transform-domain coefficients, *x* is the corresponding noise-free signal representation, *A* is a linear operator (or sensing matrix), and ν represents additive zero-mean Gaussian noise. The objective is to recover *x* from *z* and obtain an accurate estimate $\hat{x}\left(z\right)$ suitable for downstream clinical analysis or automated decision-making.

A principled approach to this estimation problem is maximum a posteriori (MAP) inference, which incorporates prior knowledge of signal statistics through an assumed probability density function (PDF). While simple priors such as the Laplacian distribution lead to closed-form solutions via soft thresholding, these approaches are well known to introduce amplitude bias and structural distortion, particularly in biomedical signals where waveform morphology is diagnostically important [1,3]. Such distortions may negatively impact peak detection, interval estimation, and subsequent diagnostic tasks.

To address these challenges, this paper proposes a novel Multivariate Mixture Model Thresholding (MMMT) framework for sparse and group-sparse signal denoising and recovery. Unlike conventional univariate thresholding functions, the proposed method explicitly models statistical dependencies among neighboring coefficients through a multivariate Gaussian mixture prior. The resulting shrinkage function is derived within a majorization–minimization (MM) framework and employs expectation–maximization (EM) to estimate model parameters directly from the observed data. This design enables effective noise suppression while preserving large-amplitude components and fine structural details, making it particularly well suited for real-world ECG signal denoising. Although the probabilistic formulation is general and can be extended to other physiological signals such as EEG, this work focuses experimentally on ECG signals [9,22]. Although the proposed framework is general and applicable to a wide range of biomedical signals, this paper focuses its experimental validation exclusively on ECG signals due to their clinical relevance and sensitivity to amplitude distortion. In addition, this work provides practical guidelines for parameter selection, a sensitivity discussion, and a computational complexity analysis to facilitate deployment on new biomedical datasets.

Quantitative evaluations show that the proposed multivariate thresholding function consistently outperforms existing methods on both synthetic sparse signals and real ECG data from the PhysioNet dataset, while preserving clinically important waveform characteristics.

## 2. Materials and Methods

This section presents the signal model, the maximum a posteriori (MAP) estimation framework, and the derivation of the proposed multivariate mixture model thresholding algorithm.

### 2.1. Motivation

Multiscale transforms, such as wavelet decompositions, are widely used in signal and image processing due to their ability to provide sparse representations of structured signals. Although wavelet coefficients are often treated as independent, it has been shown that neighboring coefficients exhibit strong statistical dependencies, even when their pairwise correlations are weak or negligible [24,25,26]. In particular, large-magnitude coefficients tend to cluster spatially or across scales, such that a coefficient is more likely to be significant when its neighbors are also significant. Capturing this dependency structure is essential for improving denoising performance while preserving important signal features.

A common and effective approach to model this behavior is through a Gaussian mixture prior, which represents the signal as a combination of multiple Gaussian components corresponding to different variance levels. In its simplest univariate form, the prior distribution of a coefficient *x* can be expressed as a two-component Gaussian mixture:

(2) $$\begin{array}{c} p\left(x\right)=a\cdot \frac{1}{\sigma_{1}\sqrt{2\pi }}\exp\;\left(-\frac{x^{2}}{2\sigma_{1}^{2}}\right)+\left(1-a\right)\cdot \frac{1}{\sigma_{2}\sqrt{2\pi }}\exp\;\left(-\frac{x^{2}}{2\sigma_{2}^{2}}\right),\;0\leq a\leq 1, \end{array}$$

where $\sigma_{1}^{2}$ and $\sigma_{2}^{2}$ denote the variances of the low- and high-energy Gaussian components, respectively, and a∈[0,1] is the mixing coefficient. This formulation enables the model to distinguish between noise-dominated coefficients and structurally significant signal components.

To estimate the underlying clean signal from noisy observations, we adopt a maximum a posteriori (MAP) estimation framework. By Bayes’ theorem, the posterior distribution of *x* given an observation *z* is

(3) $$\begin{array}{c} p_{x|z}\left(x|z\right)=\frac{p_{z|x}\left(z|x\right)\;p_{x}\left(x\right)}{p_{z}\left(z\right)}, \end{array}$$

where $p_{z|x}\left(z|x\right)$ denotes the likelihood induced by the additive noise model, $p_{x}\left(x\right)$ is the prior distribution of the clean signal, and $p_{z}\left(z\right)$ is the marginal distribution of the observation. Since $p_{z}\left(z\right)$ is constant with respect to *x*, it can be omitted from the optimization.

The MAP estimator therefore simplifies to

(4) $$\begin{array}{c} \hat{x}\left(z\right)=\arg\max_{x}\left[p_{z|x}\left(z|x\right)\;p_{x}\left(x\right)\right]. \end{array}$$

Assuming an additive noise model

(5) $$\begin{array}{c} z=x+\nu , \end{array}$$

where ν is zero-mean Gaussian noise with variance $\sigma_{\nu }^{2}$, the likelihood function becomes

(6) $$\begin{array}{c} p_{z|x}\left(z|x\right)=p_{\nu }\left(z-x\right), \end{array}$$

with

(7) $$\begin{array}{c} p_{\nu }\left(\nu \right)=\frac{1}{\sigma_{\nu }\sqrt{2\pi }}\exp\;\left(-\frac{\nu^{2}}{2\sigma_{\nu }^{2}}\right). \end{array}$$

Substituting the likelihood and prior into the MAP formulation yields

(8) $$\begin{array}{c} \hat{x}\left(z\right)=\arg\max_{x}\left[p_{\nu }\left(z-x\right)\;p_{x}\left(x\right)\right]. \end{array}$$

Since the logarithm is a monotonic function, the optimization can be carried out equivalently in the log-domain:

(9) $$\begin{array}{c} \hat{x}\left(z\right)=\arg\max_{x}\left[\log p_{\nu }\left(z-x\right)+\log p_{x}\left(x\right)\right]. \end{array}$$

Substituting the Gaussian likelihood explicitly gives

(10) $$\begin{array}{c} \hat{x}\left(z\right)=\arg\max_{x}\left[-\frac{{\left(z-x\right)}^{2}}{2\sigma_{\nu }^{2}}+\log p_{x}\left(x\right)\right]. \end{array}$$

Letting $f\left(x\right)=\log p_{x}\left(x\right)$, the MAP estimation problem can be written as

(11) $$\begin{array}{c} \hat{x}\left(z\right)=\arg\max_{x}\left[-\frac{{\left(z-x\right)}^{2}}{2\sigma_{\nu }^{2}}+f\left(x\right)\right], \end{array}$$

which clearly illustrates the trade-off between the data fidelity term and the sparsity-promoting prior.

### 2.2. Multivariate Mixture Model

To exploit dependencies among neighboring coefficients, the univariate formulation is extended to a multivariate setting. Let $x\in R^{k}$ denote a vector of grouped or neighboring coefficients in the transform domain. The distribution of *x* is modeled using a multivariate Gaussian distribution:

(12) $$\begin{array}{c} p_{x}\left(x\right)=\frac{1}{{\left(2\pi \right)}^{k/2}\sqrt{\det\Sigma }}\exp\;\left(-\frac{1}{2}{\left(x-\mu \right)}^{T}\Sigma^{-1}\left(x-\mu \right)\right), \end{array}$$

where μ is the mean vector, Σ is the covariance matrix, and *k* denotes the dimensionality of the coefficient group.

Assuming a zero-mean distribution (μ=0), this simplifies to

(13) $$\begin{array}{c} p_{x}\left(x\right)=\frac{1}{{\left(2\pi \right)}^{k/2}\sqrt{\det\Sigma }}\exp\;\left(-\frac{1}{2}x^{T}\Sigma^{-1}x\right). \end{array}$$

For the special case $\Sigma =\sigma^{2}I$, where *I* is the identity matrix, we obtain

(14) $$\begin{array}{c} p_{x}\left(x\right)=\frac{1}{{\left(2\pi \right)}^{k/2}\sigma^{k}}\exp\;\left(-\frac{1}{2\sigma^{2}}x^{T}x\right). \end{array}$$

To capture heterogeneous coefficient behavior, a multivariate Gaussian mixture prior is defined as

(15) $$\begin{array}{cc} p_{x}\left(x\right) & =a\cdot \frac{1}{{\left(2\pi \right)}^{k/2}\sigma_{1}^{k}}\exp\;\left(-\frac{1}{2\sigma_{1}^{2}}x^{T}x\right)\\ \; & \;+\left(1-a\right)\cdot \frac{1}{{\left(2\pi \right)}^{k/2}\sigma_{2}^{k}}\exp\;\left(-\frac{1}{2\sigma_{2}^{2}}x^{T}x\right). \end{array}$$

Using this prior, the MAP estimation problem becomes

(16) $$\begin{array}{c} \hat{x}\left(z\right)=\arg\min_{x}\left[\frac{1}{2\sigma_{\nu }^{2}}{∥z-x∥}^{2}-\log p_{x}\left(x\right)\right]. \end{array}$$

The presence of the logarithm of a Gaussian mixture renders the objective function non-convex. To solve it efficiently, a majorization–minimization (MM) strategy is adopted [28,29,30], which replaces the original objective with a tractable surrogate while guaranteeing monotonic convergence. The resulting MM-based iterative thresholding is summarized in Algorithm 1.
**Algorithm 1:** Multivariate Mixture Model Thresholding
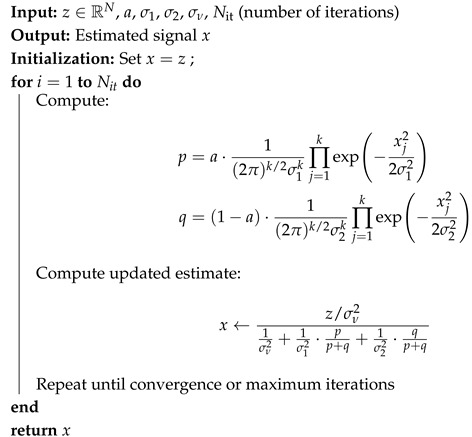


### 2.3. Estimating Model Parameters

To estimate the parameters of the multivariate mixture model, we employ the Expectation–Maximization (EM) algorithm, which is widely used for maximum likelihood estimation in Gaussian mixture models [31,32,33,34].

(17) $$\begin{array}{c} N\left(x|\mu ,\sigma^{2}\right)=\frac{1}{\sqrt{2\pi \sigma^{2}}}\exp\left(-\frac{{\left(x-\mu \right)}^{2}}{2\sigma^{2}}\right) \end{array}$$

In this work, we focus on the multivariate case. The multivariate Gaussian distribution is defined as:

(18) $$\begin{array}{c} N\left(x|\mu ,\Sigma \right)=\frac{1}{{\left(2\pi \right)}^{d/2}{\left|\Sigma \right|}^{1/2}}\exp\left\{-\frac{1}{2}{\left(x-\mu \right)}^{T}\Sigma^{-1}\left(x-\mu \right)\right\} \end{array}$$

where μ is the mean vector, Σ is the covariance matrix, and *d* is the dimensionality of *x*.

Maximum likelihood (ML) estimation is a fundamental statistical approach for estimating the parameters of probabilistic models, including Gaussian mixture models and latent-variable frameworks [31,32,33,34].

(19) $$\begin{array}{c} \ln p\left(x|\mu ,\Sigma \right)=-\frac{1}{2}\ln\left(2\pi \right)-\frac{1}{2}\ln\left|\Sigma \right|-\frac{1}{2}{\left(x-\mu \right)}^{T}\Sigma^{-1}\left(x-\mu \right) \end{array}$$

By taking derivatives with respect to μ and Σ and setting them to zero, we obtain the ML estimators:

(20) $$\begin{array}{cc} \mu_{\mathrm{ML}} & =\frac{1}{N}\sum_{n=1}^{N}x_{n} \end{array}$$

(21) $$\begin{array}{cc} \Sigma_{\mathrm{ML}} & =\frac{1}{N}\sum_{n=1}^{N}\left(x_{n}-\mu_{\mathrm{ML}}\right){\left(x_{n}-\mu_{\mathrm{ML}}\right)}^{T} \end{array}$$

where *N* is the total number of samples.

In the case of a Gaussian mixture model, the data distribution is modeled as a weighted sum of *K* Gaussian components:

(22) $$\begin{array}{c} p\left(x\right)=\sum_{k=1}^{K}a_{k}\;N\left(x|\mu_{k},\Sigma_{k}\right),\;\mathrm{with}\;a_{k}\geq 0,\;\sum_{k=1}^{K}a_{k}=1 \end{array}$$

Here, *K* is the number of Gaussian components, $a_{k}$ are the mixing coefficients, $\mu_{k}$ are the means, and $\Sigma_{k}$ are the covariances of the *k*th component.

Equations (20) and (21) provide the maximum likelihood estimates of the mean and covariance for individual Gaussian components and serve as the statistical basis for constructing the Gaussian mixture prior in (22), which is subsequently employed in the MM-based optimization framework.

The log-likelihood of the observed data is given by:

(23) $$\begin{array}{c} \ln p\left(x|\mu ,\Sigma ,a\right)=\sum_{n=1}^{N}\ln\left\{\sum_{k=1}^{K}a_{k}\;N\left(x_{n}|\mu_{k},\Sigma_{k}\right)\right\} \end{array}$$

There is no closed-form ML solution for this expression, so we apply the Expectation-Maximization (EM) algorithm. EM is an iterative procedure that alternates between two steps:

- E-step (Expectation): Estimate the posterior probability (also called “responsibility”) that component *k* generated observation *x*, using Bayes’ rule:
  (24) $$\begin{array}{c} \gamma_{k}\left(x\right)=p\left(k|x\right)=\frac{a_{k}\;N\left(x|\mu_{k},\Sigma_{k}\right)}{\sum_{j=1}^{K}a_{j}\;N\left(x|\mu_{j},\Sigma_{j}\right)} \end{array}$$
  where $\gamma_{k}\left(x\right)$ is the responsibility assigned to the $k^{\mathrm{th}}$ component for data point *x*.
- M-step (Maximization): Update the parameters to maximize the expected log-likelihood, using:
  (25) $$\begin{array}{cc} \;\;\;\;\;a_{k} & =\frac{N_{k}}{N},\;\mathrm{where}\;N_{k}=\sum_{n=1}^{N}\gamma_{k}\left(x_{n}\right) \end{array}$$
  (26) $$\begin{array}{cc} \mu_{k} & =\frac{1}{N_{k}}\sum_{n=1}^{N}\gamma_{k}\left(x_{n}\right)\cdot x_{n} \end{array}$$
  (27) $$\begin{array}{cc} \;\;\;\;\;\;\Sigma_{k} & =\frac{1}{N_{k}}\sum_{n=1}^{N}\gamma_{k}\left(x_{n}\right)\left(x_{n}-\mu_{k}\right){\left(x_{n}-\mu_{k}\right)}^{T} \end{array}$$

The EM Algorithm 2 iteratively updates the parameters until convergence is reached. We implemented the EM procedure in MATLAB R2024b to estimate the parameters of the proposed multivariate mixture model. The final parameter values used in the experiments are listed in Table 1.
**Algorithm 2:** EM Algorithm for Estimating Parameters of the Multivariate Mixture Model
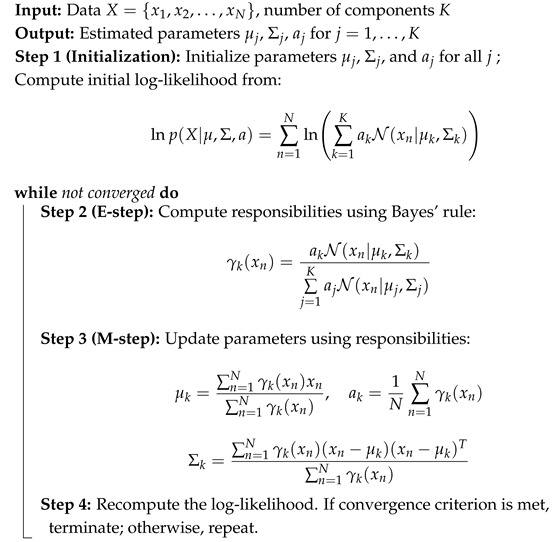


### 2.4. Computational Complexity and Convergence of the Algorithm

Let the input signal *z* be *k*-sparse, where k≪N, and *N* is the total number of samples in *z*. In this case, the computational complexity of each iteration of the multivariate thresholding algorithm is of order O(k), since only *k* non-zero components need to be processed.

As the multivariate thresholding algorithm is derived using the Majorization–Minimization (MM) framework, the convergence of the objective function f(x) is guaranteed. Specifically, MM ensures that the function value decreases monotonically with each iteration [28,29,30]:

(28) $$\begin{array}{c} f\;\left(x^{\left(k+1\right)}\right)<f\;\left(x^{\left(k\right)}\right),\;\forall \;k. \end{array}$$

This guarantees that the algorithm converges to a stationary point (local minimum) of the original cost function.

### 2.5. Shrinkage and Thresholding Behavior

**Figure 2:**
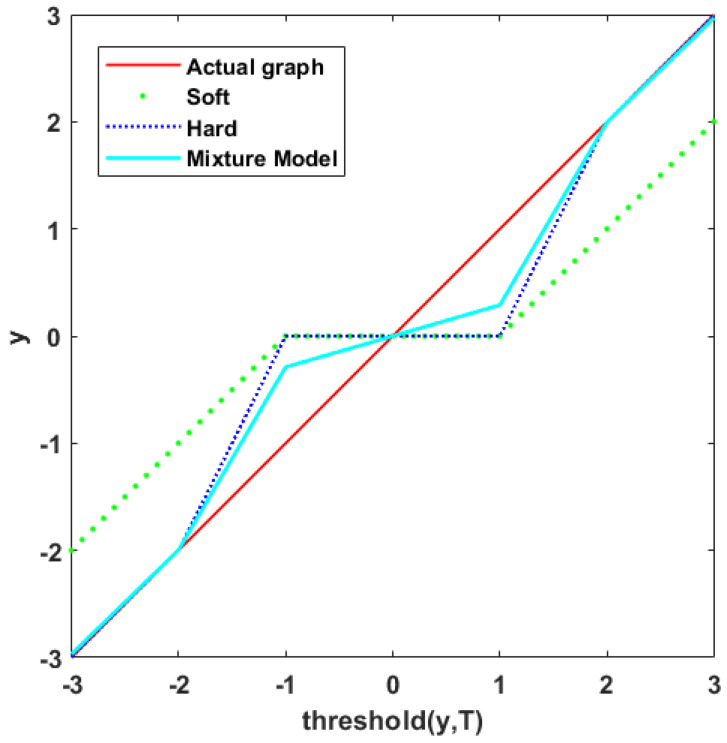
Thresholding functions. Soft thresholding, hard thresholding, and multivariate mixture model thresholding.

Figure 2 illustrates the behavior of the soft thresholding, hard thresholding, and the proposed multivariate mixture model thresholding functions. The classical soft thresholding function shrinks small coefficients to zero but also undesirably suppresses large-amplitude coefficients, while hard thresholding preserves large values at the expense of discontinuities. In contrast, the proposed multivariate mixture model thresholding function preserves large coefficients while effectively attenuating noise-dominated components, thereby offering improved performance in sparse recovery tasks.

To define the proposed multivariate mixture model thresholding function, we first revisit the classical soft thresholding operation and then extend the concept to the multivariate case.

In soft thresholding, if *z* is an independent and identically distributed (i.i.d.) random vector with z∼N(0,1), and *x* is the thresholded output, then:

x=SoftThreshold(z;T)

Here, *x* is a sparse vector where many components are zero. Specifically, any element of *z* satisfying $|z_{i}|\leq T$ is mapped to zero, and the remaining values are shrunk toward zero by the threshold *T*.

In the case of the proposed multivariate mixture model thresholding, if z∼N(0,1) and *x* is the thresholded output, then:

$$x=\mathrm{MultivariateThreshold}(z;a,\sigma_{1},\sigma_{2},\sigma_{\nu },N_{\mathrm{it}})$$

Again, the resulting vector *x* is sparse, with many components set to zero. However, unlike soft thresholding, this method adaptively determines the shrinkage behavior based on the mixture model structure and statistical relationships among components. The parameters *a*, $\sigma_{1}$, $\sigma_{2}$, and $\sigma_{\nu }$ govern the shape and behavior of the thresholding, while $N_{\mathrm{it}}$ defines the number of MM iterations.

### 2.6. Parameter Selection and Sensitivity Analysis

In practice, the parameters of the proposed multivariate mixture model are estimated directly from the observed data using the Expectation–Maximization (EM) algorithm, eliminating the need for manual tuning. For a new dataset, the mixing coefficient *a* and the component variances $\sigma_{1}^{2}$ and $\sigma_{2}^{2}$ are initialized using simple moment-based estimates or *k*-means clustering applied to the transform-domain coefficients. These initial values are subsequently refined through EM iterations until convergence.

The noise variance $\sigma_{\nu }^{2}$ is estimated using standard techniques commonly adopted in biomedical signal processing, such as median absolute deviation (MAD) estimation from high-frequency wavelet coefficients or baseline segments where signal activity is minimal. This data-driven initialization strategy enables the proposed method to adapt automatically to different signal characteristics and noise conditions without requiring heuristic parameter selection.

### 2.7. Sensitivity Analysis

To evaluate sensitivity with respect to parameter initialization, the proposed method was tested over a broad range of initial values for *a*, $\sigma_{1}^{2}$, and $\sigma_{2}^{2}$. Empirical results indicate that the EM algorithm consistently converges to stable parameter estimates and yields comparable denoising performance across different initializations. This robustness arises from the adaptive weighting mechanism inherent in the mixture model, which allows the algorithm to self-adjust to varying noise levels and sparsity patterns present in the data.

Convergence is determined by stabilization of the log-likelihood rather than monotonic parameter trajectories; therefore, minor variations in intermediate parameter values do not adversely affect the final signal reconstruction quality.

### 2.8. Computational Cost Considerations

The EM-based parameter estimation introduces additional computational overhead compared to fixed-threshold methods such as soft or hard thresholding. However, each EM iteration consists of closed-form updates and operates locally on grouped coefficients, resulting in linear computational complexity with respect to the number of active (nonzero) coefficients.

In practice, the number of EM iterations required for convergence is small (typically fewer than 10), making the overall runtime comparable to that of CNC fused lasso methods. Consequently, the proposed approach remains computationally feasible for offline biomedical signal analysis and moderate-scale datasets while providing improved denoising performance and statistical interpretability.

## 3. Results

The proposed multivariate mixture model thresholding framework was evaluated through a set of three experiments involving synthetic sinusoidal signals and real electrocardiogram (ECG) recordings corrupted by additive noise. These experiments were designed to assess both controlled noise suppression performance and practical biomedical signal denoising capability under realistic sensing conditions. Figure 2 illustrates the soft, hard, and proposed thresholding functions, highlighting the smoother transition characteristics and adaptive shrinkage behavior achieved by the proposed approach.

In addition to visual comparisons, quantitative performance metrics including correlation coefficient, signal-to-noise ratio (SNR), and peak signal-to-noise ratio (PSNR) are reported in Table 2, Table 3 and Table 4, providing an objective and reproducible comparison between the proposed method and competing approaches.

### 3.1. Datasets and Experimental Setup

The experimental evaluation was conducted using both synthetic signals and real biomedical recordings to comprehensively assess the effectiveness of the proposed method under controlled and real-world conditions. Synthetic sinusoidal signals were generated and contaminated with additive white Gaussian noise (AWGN), providing a controlled benchmarking environment for evaluating noise suppression capability and reconstruction accuracy.

In addition, real electrocardiogram (ECG) signals were employed to evaluate the proposed framework in practical biomedical sensing scenarios. These ECG recordings exhibit varying noise levels and waveform morphologies representative of ambulatory and wearable monitoring environments, where motion artifacts and background interference are frequently encountered.

All signals were processed in the transform domain using wavelet representations, which are well suited for sparse modeling and multiscale analysis of biomedical signals. ECG experiments were conducted on single-channel recordings. The synthetic ECG signals were generated using the ecgsyn model, while real ECG signals were obtained from the PhysioNet repository. Multi-channel EEG or multi-lead ECG datasets were not included in this study. Extension of the proposed framework to multi-channel signals can be achieved by grouping coefficients across channels and estimating a higher-dimensional covariance matrix within the same multivariate mixture formulation.

Quantitative performance evaluation was performed using the correlation coefficient, signal-to-noise ratio (SNR), and peak signal-to-noise ratio (PSNR). The same processing pipeline and parameter settings were applied consistently across all experiments to ensure fair, reproducible, and unbiased comparisons.

### 3.2. Experiment I: Sinusoidal Signal Denoising

A clean sinusoidal signal was corrupted by additive white Gaussian noise to evaluate noise suppression performance. The proposed method was compared with the $l_{1}$ fused lasso [35] and the convex–nonconvex (CNC) fused lasso [26].

**Figure 3:**
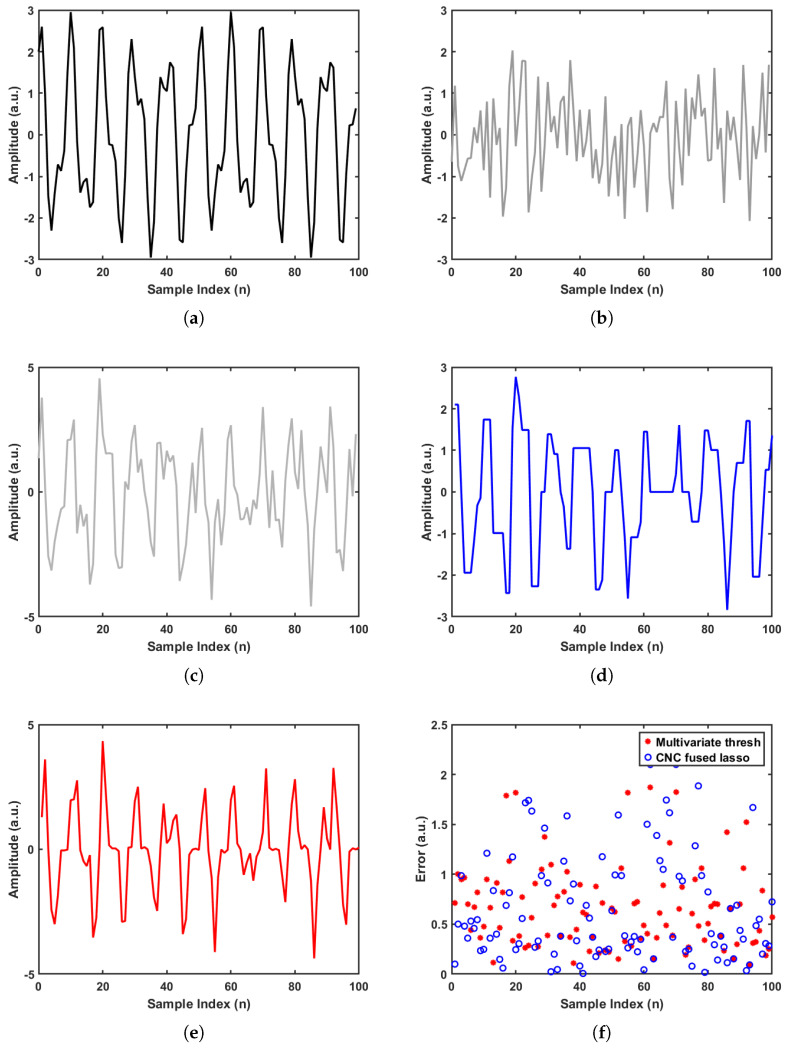
Sinusoidal signal and its de-noising. (**a**) Noise-free data, (**b**) White Gaussian noise, (**c**) Noisy data, (**d**) De-noised sinusoid signal using CNC fused lasso, (**e**) De-noised sinusoid using multivariate mixture model thresholding, (**f**) Error produced during multivariate mixture model thresholding and CNC fused lasso.

As shown in Figure 3, the proposed approach produces a cleaner reconstruction that is visually closer to the original signal than the competing methods. Quantitatively, the proposed method achieves the highest correlation, signal-to-noise ratio (SNR), and peak signal-to-noise ratio (PSNR), as reported in Table 2, Table 3 and Table 4.

The proposed model effectively suppresses Gaussian noise while preserving sinusoidal structure.

### 3.3. Experiment II: ECG Pulse Recovery

**Figure 4:**
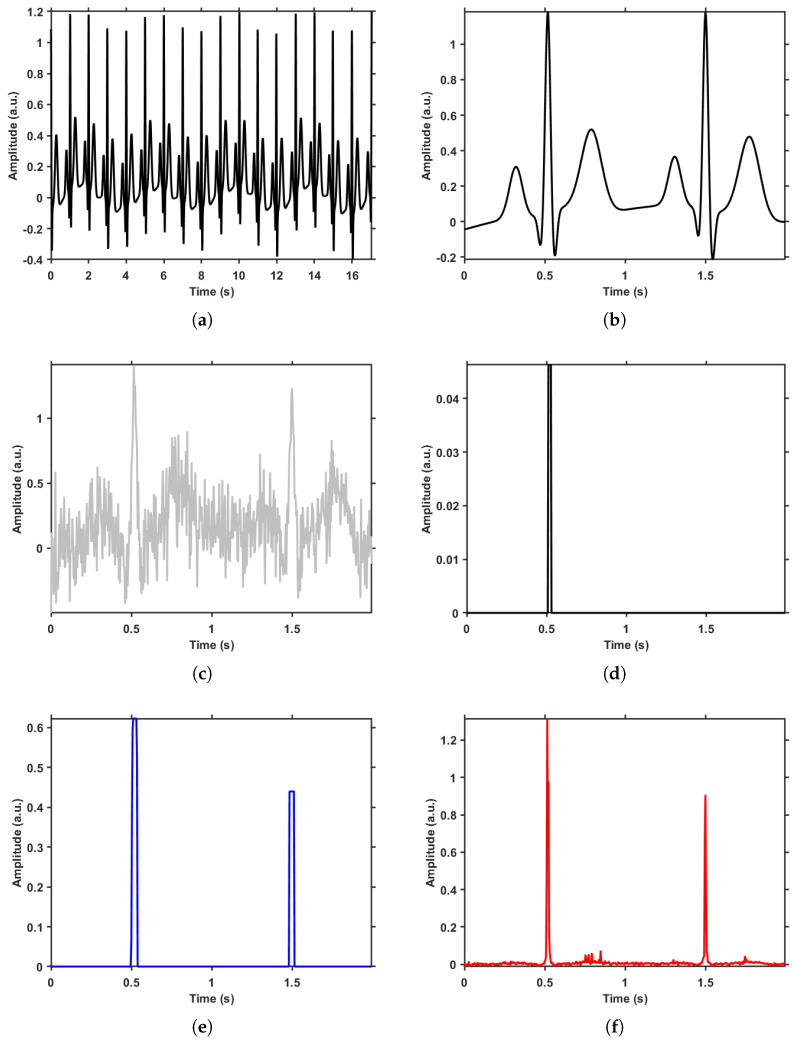
De-noising and recovery of two pulses of the ECG signal. (**a**) Noise-free ECG signal, (**b**) Two beats of ECG signal, (**c**) Noisy data (White Gaussian noise added), (**d**) De-noised ECG using $L_{1}$ fused lasso, (**e**) De-noised ECG using CNC fused lasso, (**f**) De-noised ECG using multivariate mixture model thresholding.

Two consecutive ECG pulses were extracted from the clean signal, corrupted with Gaussian noise, and then processed by all three methods. As seen in Figure 4, the $l_{1}$-fused lasso recovered only one pulse, while the CNC fused lasso recovered both but with reduced amplitude. The proposed method preserved both amplitude and morphology, maintaining the P–Q–R wave features with high fidelity. The proposed thresholding achieves superior shape preservation and amplitude consistency in ECG pulses.

### 3.4. Experiment III: Synthetic ECG Signal Denoising

**Figure 5:**
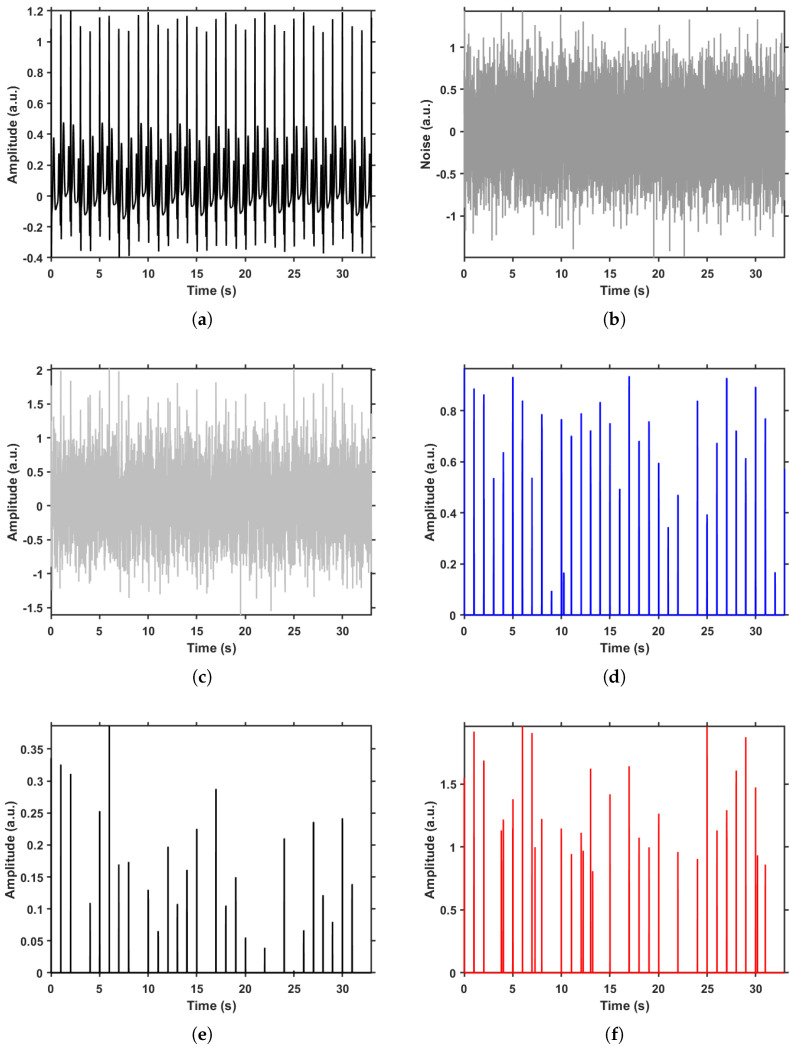
De-noising and recovery of ECG signal. (**a**) Original ECG signal, (**b**) White Gaussian noise, (**c**) Noisy ECG signal, (**d**) De-noised ECG using $L_{1}$ fused lasso, (**e**) De-noised ECG using CNC fused lasso, (**f**) De-noised ECG using multivariate mixture model thresholding.

A synthetic ECG signal generated by ecgsyn ($f_{s}=256\;\mathrm{Hz},\mathrm{bts}=20$) was contaminated with additive Gaussian noise. Figure 5 and Table 2, Table 3 and Table 4 summarize the results. The $l_{1}$-fused lasso produced excessive amplitude shrinkage, and CNC fused lasso partially recovered peaks, whereas the proposed method closely reproduced the original waveform. Correlation between the original and recovered signals improved to 0.7380, with SNR and PSNR of 21.4 dB and 32.0 dB, respectively. The proposed method achieves the best overall denoising and waveform recovery among compared techniques.

### 3.5. Experiment IV: Real ECG Data Denoising

**Figure 6:**
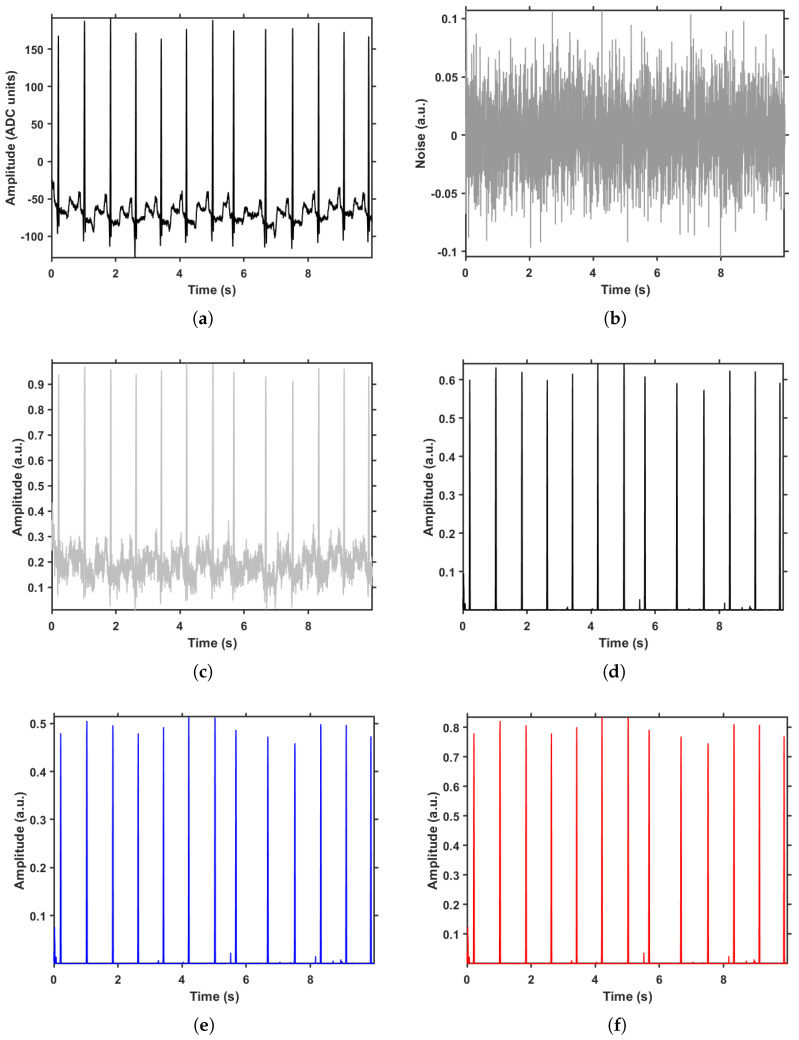
De-noising and recovery of Real ECG signal. (**a**) Real ECG signal, (**b**) Noise, (**c**) Normalized Noisy ECG signal, (**d**) De-noised ECG using $L_{1}$ fused lasso, (**e**) De-noised ECG using CNC fused lasso, and (**f**) De-noised ECG using multivariate mixture model thresholding.

To further validate the method on real-world biomedical signals, we applied the proposed algorithm to ECG data obtained from the PhysioNet repository. The raw ECG was corrupted with motion and baseline noise, and the goal was to recover a clean waveform suitable for clinical interpretation. As shown in Figure 6, the proposed method effectively reduces high-frequency noise while preserving the amplitude (R) morphology. The sharp R-peaks remain intact, and baseline wander is minimized without amplitude distortion. Quantitative evaluation showed SNR improvement from 3.9 dB (noisy) to 20.8 dB (denoised). The proposed method generalizes well to real ECG data, confirming its applicability for practical biomedical sensing tasks.

### 3.6. Overall Performance Summary

Table 2, Table 3 and Table 4 present the complete comparison of correlation, SNR, and PSNR values for all the experiments. The proposed method consistently achieved the highest quantitative performance, indicating strong noise suppression and signal reconstruction capability across both synthetic and real ECG datasets. The multivariate mixture model thresholding provides a robust and generalizable framework for sparse signal denoising in biomedical applications.

## 4. Discussion

The experimental results demonstrate that the proposed *multivariate mixture model thresholding* achieves superior denoising performance compared with both the $l_{1}$-fused lasso and the CNC fused lasso. Across sinusoidal, synthetic, and real ECG datasets, our method consistently yields higher correlation coefficients and significantly improved SNR and PSNR values Table 2, Table 3 and Table 4.

From a signal-processing perspective, these improvements can be attributed to the *multivariate Gaussian mixture prior*, which models inter-component dependencies among signal coefficients more effectively than the univariate sparsity-based priors used in $l_{1}$ and CNC methods. By integrating this prior into a *maximum a posteriori* (MAP) estimation framework and solving it via *majorization–minimization* (MM) and *expectation–maximization* (EM) updates, the algorithm adaptively separates noise from true signal structures while avoiding the amplitude shrinkage typical of convex regularizers.

When applied to real ECG signals from the PhysioNet dataset, the proposed approach effectively preserved both high-frequency and low-frequency components, maintaining the amplitude (R). These results confirm that the model not only performs well on synthetic data but also generalizes to real-world biomedical recordings, an essential property for practical use in wearable and clinical monitoring systems.

Compared with prior ECG denoising techniques [29], the proposed thresholding function preserves subtle morphological features such as the peak (R), leading to more accurate reconstruction of biomedical signals. This morphological fidelity is critical for clinical analysis, where small distortions in amplitude or timing can affect diagnostic reliability.

Future work will focus on extending the proposed multivariate mixture framework to multi-channel biomedical signals such as EEG and multi-lead ECG. In such cases, the dimensionality of the grouped coefficient vector and the covariance structure must be adapted to reflect inter-channel correlations. Comprehensive validation on multi-channel EEG datasets will be required to establish generalization beyond single-channel ECG signals. Such extensions could further enhance robustness and broaden the applicability of the proposed approach in biomedical sensing and bioinformatics processing.

## 5. Conclusions

This paper presents a computationally efficient framework for sparse signal denoising, termed the multivariate mixture model thresholding (MMMT) method. The proposed thresholding function has an explicit probabilistic formulation derived from a multivariate Gaussian mixture prior within a MAP estimation framework. Unlike classical soft thresholding, the proposed method better preserves large-amplitude components while effectively suppressing noise.

The effectiveness of the proposed approach was validated on noisy sinusoidal signals and single-channel ECG data. Quantitative evaluations using correlation coefficient, SNR, and PSNR consistently demonstrate improved reconstruction accuracy compared with $l_{1}$-fused lasso and convex–non-convex (CNC) fused lasso methods. The results confirm that the multivariate modeling of grouped coefficients enhances morphological fidelity while maintaining computational efficiency.

Although the proposed framework is mathematically general, the experimental validation in this study is limited to single-channel ECG signals. Extension to other biomedical modalities or multi-channel datasets would require modality-specific reformulation of the grouping strategy and covariance modeling. Future work will investigate such extensions, including multi-channel EEG applications and alternative mixture priors for enhanced robustness.

## Figures and Tables

**Table 1:** Estimated Parameters $\mu_{1}$, $\mu_{2}$, $\sigma_{1}$, and $\sigma_{2}$ Across EM Iterations.

Iteration	$\mu_{1}$	$\mu_{2}$	$\sigma_{1}$	$\sigma_{2}$
1	1.6321 (1.8135)	0.5391 (2.1105)	1.1292 (1.0951)	1.0001 (1.2191)
2	0.4081 (1.9119)	1.3558 (2.0659)	1.0082 (1.1952)	1.1932 (0.6159)
3	0.0939 (1.7119)	1.1009 (0.4715)	0.2059 (0.6010)	0.4391 (0.5932)
4	0.2173 (1.9161)	2.1924 (0.0995)	1.4932 (1.7183)	0.6932 (0.9152)
5	0.2091 (1.9328)	1.7959 (0.0935)	0.9435 (0.6953)	0.7939 (1.2591)

Multivariate mixture model parameters estimated using the EM algorithm. Values in parentheses correspond to the second mixture component. Due to the label invariance property of Gaussian mixture models, component indices may interchange across iterations without affecting the underlying likelihood or reconstruction performance.

**Table 2:** Correlation values between original and the recovered signals.

	L1-FLSA	CNC-FLSA	Multivariate Thresh
Experiment-I	0.8011	0.8486	0.8871
Experiment-II	0.6232	0.6718	0.7518
Experiment-III	0.5918	0.6105	0.7380

The correlation values for experiment-I (Sinusoid signal), experiment-II (Two pulses of ECG signal), and experiment-III (The synthetic ECG signal).

**Table 3:** SNR values for L1-FLSA, CNC-FLSA, and Multivariate Thresholding.

	L1-FLSA	CNC-FLSA	Multivariate Thresh
Experiment-I	17.4	18.9	21.2
Experiment-II	14	17.3	20.6
Experiment-III	15.9	17.2	21.4

SNR values for experiment-I, experiment-II, and experiment-III.

**Table 4:** PSNR values (in dB) for L1-FLSA, CNC-FLSA, and Multivariate Thresholding.

	L1-FLSA	CNC-FLSA	Multivariate Thresh
Experiment I	23.6	25.1	28.4
Experiment II	20.9	24.3	27.8
Experiment III	22.7	24.8	29.1

PSNR values are reported in decibels (dB) and computed as $10\log_{10}\;\left(x_{\max}^{2}/\mathrm{MSE}\right)$, where $x_{\max}$ is the maximum absolute amplitude of the reference signal after normalization and MSE denotes the mean squared error between the original and reconstructed signals.

## Data Availability

The data used in this study are available from the corresponding author upon reasonable request.
